# Case Report: Altered NK Cell Compartment and Reduced CXCR4 Chemotactic Response of B Lymphocytes in an Immunodeficient Patient With HPV-Related Disease

**DOI:** 10.3389/fimmu.2022.799564

**Published:** 2022-01-26

**Authors:** Margherita Doria, Giusella M. F. Moscato, Silvia Di Cesare, Gigliola Di Matteo, Mayla Sgrulletti, Françoise Bachelerie, Viviana Marin-Esteban, Viviana Moschese

**Affiliations:** ^1^ Research Unit of Primary Immunodeficiency, Bambino Gesù Children’s Hospital, Istituto di Ricerca e Cura a Carattere Scientifico (IRCCS), Rome, Italy; ^2^ Infectious Diseases Unit, Policlinico Tor Vergata, University of Tor Vergata, Rome, Italy; ^3^ Department of Medicine of Systems, University of Tor Vergata, Rome, Italy; ^4^ Pediatric Immunopathology and Allergology Unit, Policlinico Tor Vergata, University of Tor Vergata, Rome, Italy; ^5^ PhD Program in Immunology, Molecular Medicine and Applied Biotechnology, University of Rome Tor Vergata, Rome, Italy; ^6^ Université Paris-Saclay, Inserm, Inflammation, Microbiome and Immunosurveillance, Clamart, France

**Keywords:** WHIM, CXCR4/CXCL12 axis, B lymphocytes, NK cells, HPV

## Abstract

The study of inborn errors of immunity (IEI) provides unique opportunities to elucidate the microbiome and pathogenic mechanisms related to severe viral infection. Several immunological and genetic anomalies may contribute to the susceptibility to develop Human Papillomavirus (HPV) pathogenesis. They include different acquired immunodeficiencies, *EVER1-2* or *CIB1* mutations underlying epidermodysplasia verruciformis (EV) syndrome and multiple IEI. Whereas EV syndrome patients are specifically unable to control infections with beta HPV, individuals with IEI show broader infectious and immune phenotypes. The WHIM (warts, hypogammaglobulinemia, infection, and myelokathexis) syndrome caused by gain-of-*CXCR4*-function mutation manifests by HPV-induced extensive cutaneous warts but also anogenital lesions that eventually progress to dysplasia. Here we report alterations of B and NK cells in a female patient suffering from cutaneous and mucosal HPV-induced lesions due to an as-yet unidentified genetic defect. Despite no detected mutations in *CXCR4*, B but not NK cells displayed a defective CXCR4-dependent chemotactic response toward CXCL12. In addition, NK cells showed an abnormal distribution with an expanded CD56^bright^ cell subset and defective cytotoxicity of CD56^dim^ cells. Our observations extend the clinical and immunological spectrum of IEI associated with selective susceptibility toward HPV pathogenesis, thus providing new insight on the immune control of HPV infection and potential host susceptibility factors.

## Introduction

More than 200 types of human papilloma virus (HPV) ordered into 5 genera have been recognized and classified as cutaneous or mucosal according to their tropism (1). The alpha genus comprises HPV types mostly associated with the development of anogenital and oropharyngeal carcinomas whereas the beta genus can be associated with cutaneous squamous cell carcinoma. Patients with EV, a rare genodermatosis due to mutations of the EVER1/EVER2/CIB1 trans-membrane channel proteins, develop persistent disseminated beta HPV-derived verrucous cutaneous lesions in sun-exposed areas that might become malignant later in life (2). These patients show normal adaptive immunity while their exclusive susceptibility to develop HPV-related disease might be linked to epithelial homeostasis (2). Recent years have seen growing evidence of recurrent and severe non-beta genus restricted HPV infections among immunosuppressed individuals with T cell primary immunodeficiency (3). Several germline mutations causing IEI, such as those affecting *CXCR4*, *GATA2*, *SASH3* and *WAS*, have been associated with disseminated cutaneous and anogenital HPV-induced pathology (3, 4).

The identification of CXCR4 gain of function mutations in the etiology of the WHIM syndrome has expanded our knowledge of the physiopathology of this receptor and its CXCL12 chemokine ligand. In WHIM, premature termination or frameshift mutations in the cytoplasmic tail of CXCR4 prevent receptor internalization/desensitization in response to CXCL12 binding, which accounts for enhancement of CXCR4 expression and chemotactic responses to CXCL12 (5–7). The exacerbated CXCR4/CXCL12 signaling notably hampers the trafficking of myeloid cells, results in the retention of hyper mature neutrophils in the bone marrow (i.e. myelokathexis) and affects both innate and adaptive immune responses (8–13). In contrast, the impact of altered CXCR4/CXCL12 axis in NK cells is still poorly defined (14–16). Over the years, WHIM-like disorders showing variations both in genotype and phenotype from the paradigmatic WHIM syndrome have been reported, also including disorders developing in the absence of *CXCR4* mutations (13). Here we report B and NK cell anomalies in a female patient with HPV-related disease for whom we have previously proposed a diagnostic hypothesis of WHIM-like syndrome with wild-type CXCR4 (17). The expression of CXCR4 in peripheral lymphocyte subsets and their CXCR4/CXCL12-dependent chemotaxis were investigated unveiling a previously unrecognized B lymphocyte defect discrepant with a WHIM-related response. Together with the identification of anomalies affecting the phenotype and the function of NK cells, our data frame a novel IEI associated with severe HPV infection.

### Case Description

**Figure 1:**
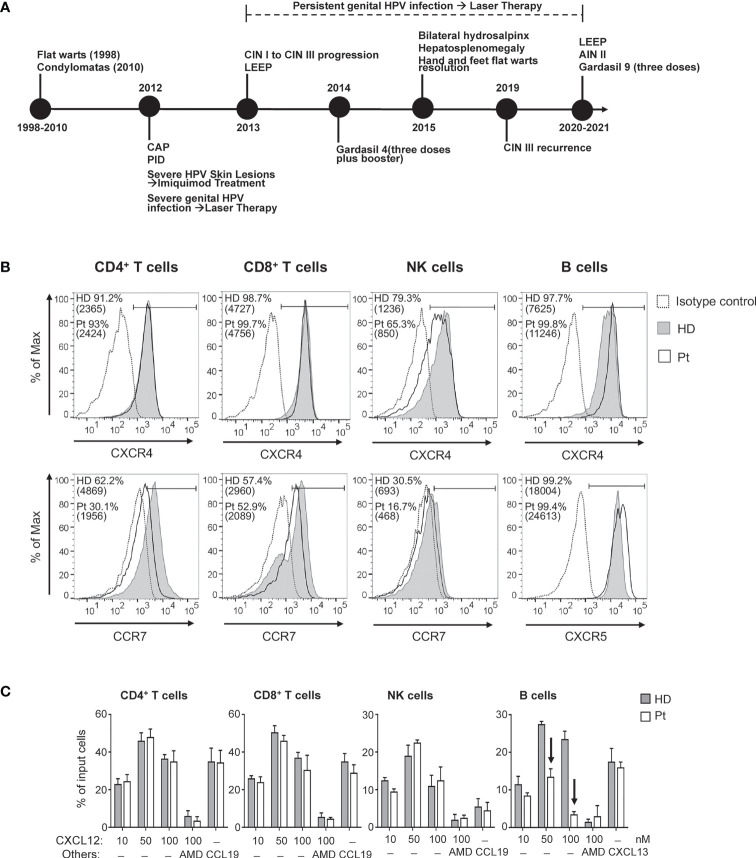
**(A)** Clinical history of the patient (CAP, community acquired pneumonia; PID, pelvic inflammatory disease; CIN, cervical intraepithelial neoplasia; AIN, anal intraepithelial neoplasia; LEEP, loop electrosurgical excision procedure). Chemokine receptor expression and chemotactic response of blood lymphocyte subsets. **(B)** Cell surface expression of CXCR4, CCR7 and CXCR5 was analyzed in CD4^+^ T, CD8^+^ T, NK, and B cells upon immunolabeling of peripheral blood cells from the patient (Pt; open histograms) and age-matched HD (filled gray histograms). The control IgG signal (dashed lines) is also shown. Values corresponding to percentage of positive cells as well as MFI (in brackets) are reported. **(C)** The chemotactic response of blood lymphocyte subsets was analyzed using whole blood samples from HD and Pt; results are expressed as percentage of input cells responding to the indicated concentrations of CXCL12, to 100 nM CCL19, or 300 nM CXCL13. AMD: AMD3100. Arrows indicate chemotactic responses notably reduced in Pt as compared with HD.

Our patient is a 32-year-old female who was described as a WHIM-like disorder at 26 years of age on the basis of dysplasia of granulocytes, recurrent infections, HPV-associated disease and B-cell lymphopenia (17). As previously reported, no family members showed HPV-susceptibility. However, one brother of four siblings died due to pneumonia at 6 months of age. The patient suffered from recurrent upper respiratory tract infections treated with antibiotic therapy, mostly in the first two decades of life. Later on, HPV-associated disease featured the clinical picture (**Figure 1A**). Also, early loss of teeth since childhood, periodontal disease and hepatosplenomegaly was present. No autoimmune manifestations were observed. Molecular genetic testing for *CXCR4* as well as for *GATA2*, *NEMO*, and *CD40L*, revealed a wild-type status. Moreover, significant data did not emerge from the analysis of a previously described Next Generation Sequencing (NGS) panel that includes well-defined IEI genes (>300) plus many candidate genes associated with critical immune pathways (18), and Whole Exome Sequencing (WES) of the patient and parents (trio). Although in the bone marrow approximately 10% of neutrophils showed typical multilobed nuclei reminiscent of myelokathexis, the lack of perivascular clusters made us describe it as dysplasia of granulocytes. Moreover, peripheral blood cell counts showed normal values of neutrophils, monocytes, and lymphocytes but high levels of eosinophils. Blood smears revealed hypo-segmented nuclei with long filaments of chromatin connecting nuclear lobes in neutrophils and eosinophils. Repeated immunophenotypic analysis of peripheral blood mononuclear cells (PBMCs), before and after lesion treatment, showed persistent B-cell lymphopenia (3%), normal distribution of T- and B-cell subsets except for a gradual increase in CD21^low^ B lymphocytes (from 13.4% to 34%). Serum immunoglobulin levels, including IgG subclasses and IgE, were within the normal range. Protective anti-pneumococcus IgG levels were detected after polysaccharide vaccine, although they tended to wane over time (17). Also, an adequate humoral and cellular response to COVID-19 vaccine was observed. Over the years, her severe foot and hand cutaneous warts continued to benefit from the treatment attempt with HPV vaccine (three doses and a booster of Gardasil 4 plus three doses of Gardasil 9), although her mucosal lesions persisted (19). Recently, she mostly suffered from severe vulvar and cervical condylomata as well as cervical intraepithelial neoplasia (grade 3), requiring several biopsies and Loop Electrosurgical Excision Procedure, twice. Anal intraepithelial lesions (grade 2) have developed, too. HPV genotyping from cervical biopsies revealed HPV types 6, 18, 51, and 58.

The cell-surface expression of CXCR4 was comparatively analyzed by flow cytometry on distinct lymphocyte subsets of our patient and an age-matched healthy donor (HD) (for detailed Materials and Methods please see **Supplementary Material**). The CXCR4 expression level on CD4^+^ and CD8^+^ T lymphocytes was similar in the patient and in HD (**Figure 1B**). Instead, patient’s CXCR4 expression was somewhat reduced on NK cells (30% lower MFI) but increased on B lymphocytes (47% higher MFI) as compared with HD cells. We also explored the expression level of CCR7, a chemokine receptor controlling the homing to secondary lymphoid tissues of naïve T and B lymphocytes and CD56^bright^ NK cells, as well as the expression level of CXCR5, controlling homing of B lymphocytes to follicular compartments on secondary lymphoid tissues (20, 21). In patient’s cells the relative expression level of CCR7 was reduced by 30 to 40% in CD4^+^ T, CD8^+^ T, and NK cells as compared with values in HD cells. Finally, relative CXCR5 expression on B lymphocytes was 37% higher in the patient as compared with HD.

The transwell migration assay showed that CD4^+^ and CD8^+^ T lymphocytes as well as NK cells derived from the patient’s blood displayed a normal chemotactic response towards CXCL12 (**Figure 1C**), despite the reduction of CXCR4 expression we observed on patient’s NK cells. The observed dose-response effect was CXCL12/CXCR4-mediated as revealed by the inhibition provided by AMD3100, a specific inhibitor of CXCR4. Similarly, the CCR7-dependent chemotactic response to CCL19 of these lymphocyte subsets was equivalent in patient- and HD-derived cells, despite the observed differences in relative CCR7 expression levels. CCR7-dependent chemotactic response of NK cells was modest in conformity with the overall low CCR7 expression level. In contrast, for the patient-derived B lymphocytes, the CXCR4-dependent chemotactic response was strongly reduced as compared to HD-derived cells, with a loss of chemotactic efficacy of 51 ± 7.6% and 80 ± 2.8% in response to 50 nM CXCL12 and 100 nM CXCL12, respectively (**Figure 1C**), in spite of increased expression levels of CXCR4 on B lymphocytes. Of note, the response of patient-derived B-lymphocytes to CXCL13, a ligand of CXCR5, was not affected as compared with HD.

**Figure 2:**
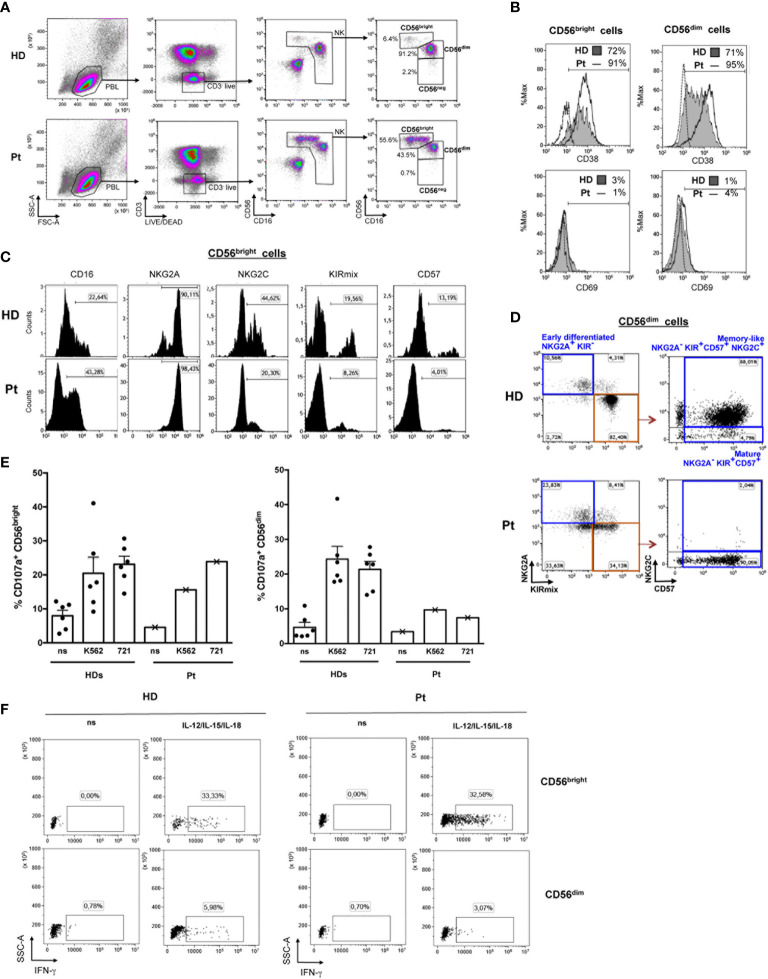
Flow cytometry-based analysis of NK cell subsets. **(A)** The gating strategy used to identify NK cells among PBMCs and measure the frequencies of CD56^bright^, CD56^dim^, and CD56^neg^ subsets is shown for the patient (Pt) and a representative HD. **(B)** The frequency of CD38^+^ and CD69^+^ cells in CD56^bright^ and CD56^dim^ NK cells from the patient (open histograms) and a representative HD (filled gray histograms) is shown together with control IgG signal (dashed lines). **(C)** Histograms depict expression of CD16, NKG2A, NKG2C, KIR (KIRmix: KIR2DL1/S1/S3/S5 and KIR2DL2/L3/S2), and CD57 on CD56^bright^ cells gated as shown in panel **(A)**. **(D)** The gating strategy used to identify different maturation subsets (Early differentiated, Mature, and Memory-like) on the basis of NKG2A, NKG2C, CD57 and KIR expression in CD56^dim^ cells gated as described in panel **(A)** is shown for the Pt and a representative CMV-seropositive HD. **(E)** Bar plots represent pattern of CD107a expression measured by flow cytometry on gated CD56^bright^ (left) and CD56^dim^ (right) NK cells of the WHIM-like patient and HDs following 6 hours culture of PBMCs with and without (non stimulated, ns) K562 or 721.221 (721) cell targets. The mean ± SD of 6 HDs is reported. **(F)** Dot plots depict intracellular IFN-γ expression in gated CD56^bright^ and CD56^dim^ NK cells following 20 hours culture in the presence or absence (ns) of IL-12, IL-15, and IL-18 of PBMCs derived from a representative HD and the WHIM-like patient. The percentage of IFN-γ^+^ cells is reported.

Patient’s NK cells had a normal frequency in peripheral blood but their subset distribution was profoundly altered (**Table 1** and **Figure 2A**). We observed a striking expansion of CD56^bright^ cells (55.6% vs. median 5.8% in HDs) at the expense of CD56^dim^ cells (43.5% vs. median 87.8% in HDs). Then, we investigated the phenotype of CD56^bright^ and CD56^dim^ subsets in our patient as compared with HDs. Both patient’s NK-cell subsets showed increased expression of the CD38 activation marker, albeit CD69 was very low as in HDs’ cells (**Figure 2B**). The vast majority of patient’s CD56^bright^ cells displayed an immature NKG2A^+^KIR^-^CD57^-^ phenotype as seen in HD (**Figure 2C**), with an overall expansion of a CD56^bright^CD16^-^NKG2A^+^NKG2C^-^KIR^-^CD57^-^ NK cell population (27.3% vs. median 2.4% of HDs) (**Table 1**). The expression of perforin and of the NKG2D, NKp46, and DNAM-1 activating receptors in patient’s CD56^bright^ and CD56^dim^ cells was normal, yet the frequency of NKp46^+^ CD56^bright^ cells was higher than in HD (97.6% vs. 78%, respectively) (**Table 2**). Moreover, we analyzed the distribution of CD56^dim^ cell maturation subsets which progress from early-differentiated (NKG2A^+^ KIR^-^) to fully mature (NKG2A^-^KIR^+^CD57^+^) cells and, eventually, to memory-like (NKG2A^-^NKG2C^+^KIR^+^CD57^+^) cells, a highly cytotoxic NK cell subset with immune adaptive properties that is expanded in CMV-seropositive individuals (23). **Figure 2D** shows that our patient had a normal frequency of early-differentiated and mature CD56^dim^ cells (**Table 1**), but lacked memory-like CD56^dim^ cells despite CMV seropositivity. Next, we analyzed the function of CD56^bright^ and CD56^dim^ NK cells by measuring their degranulation activity (i.e. CD107a expression) against HLA class I-devoid cell targets (K562 and 721.221 cells) as well as their capacity to produce IFN-γ upon stimulation with IL-12, IL-15, and IL-18 cytokines, an NK-cell activity primarily exerted by CD56^bright^ cells. As shown in **Figures 2E, F**, patient’s CD56^bright^ cells were able to degranulate and accumulate IFN-γ in response to cytokines similarly to HD cells. In contrast, patient’s CD56^dim^ NK cells were impaired in their capacity to degranulate against cell targets as compared to HDs.

**Table 1:** Distribution of NK cells.

	HDs25-37y (n=9)	Pt32y
NK		
% of PBL	7.5 (6.1-8)	7.7
NK cell subsets (%)		
CD56^bright^	5.8 (3.2-9.3)	55.6
CD56^dim^	87.8 (80.5-88.5)	43.5
CD56^neg^	6.5 (6.4-8.8)	0.6
CD56^bright^CD16^-^NKG2A^+^NKG2C^-^CD57^-^	2.4 (1.5-3.9)	27.3
CD56^dim^ subsets (%)		
NKG2A^+^KIR^-^ (early differentiated)	33.6 (12.4-46.2)	23.8
NKG2A^-^KIR^+^CD57^+^ (mature)	31.3 (13.7-44.4)	30.7
NKG2A^-^KIR^+^CD57^+^NKG2C^+^ (memory-like)	2.8 (0.5-42.4)*	0.7

Values are indicated as median (IQR, interquartlile range).

*1.7(0.1-6) and 25(2.5-80) in CMV-seronegative and -seropositive subjects, respectively (22).

y, years.

**Table 2:** NK-cell activating receptors and perforin profile.

	HDs25-37y (n=9)	Pt32y
**CD56^bright^**		
NKG2D		
% of NKG2D^+^	73.1 (66.8-79.8)	49.9
NKG2D MFI	3626 (1386-5397)	1381
NKp46		
% of NKp46^+^	78 (64.5-87.8)	97.6
NKp46 MFI	3800 (2785-4434)	10232
DNAM-1		
% of DNAM-1^+^ cells	93.7 (77.4-94.1)	99.3
DNAM-1 MFI	14157 (4380-14994)	7434
Perforin		
% of Perforin^+^ cells	100 (100-100)	99.7
Perforin MFI	18680 (14092-37817)	19054
**CD56^dim^**		
NKG2D		
% of NKG2D^+^	62.5 (53.9-79.7)	66.3
NKG2D MFI	2358 (1601-4404)	1277
NKp46		
% of NKp46^+^	83.7 (77.3-88.5)	88.8
NKp46 MFI	4100 (2960-5441)	5949
DNAM-1		
% of DNAM-1^+^ cells	75.4 (71.9-94.5)	91.8
DNAM-1 MFI	5629 (5026-7321)	6159
Perforin		
% of Perforin^+^ cells	99 (97.2-100)	97.6
Perforin MFI	59279 (31607-100561)	54597

Values are indicated as median (IQR, interquartlile range).

y, years; NKG2D, Natural Killer Group 2 member D; NKp46, Natural Killer cell p46-related protein; DNAM-1, DNAX Accessory Molecule 1.

## Discussion

Individuals with IEI may suffer of increased infectious susceptibility either to a specific pathogen or multiple pathogens (24). Here we provide original data on a patient previously proposed as a potential WHIM-like case with disseminated and persistent mucocutaneous HPV infection, recurrent upper respiratory tract infections, B-cell lymphopenia and dysplasia of granulocytes, while in a context of wild-type CXCR4 and normal peripheral neutrophil and monocyte counts (17, 19). In line with previous observations made in typical WHIM patients, our patient showed B-cell lymphopenia as prominent immunologic alteration and poor maintenance of memory response to vaccines. In the present study further immunological aspects were investigated in this patient. We found that, as compared with HD, CXCR4 was expressed at normal levels in patient’s CD4^+^ and CD8^+^ T lymphocytes, slightly reduced in NK cells yet it was increased in B lymphocytes. Moreover, we showed that the chemotactic response towards CXCL12 was maintained in patient’s T and NK cells but was strongly reduced in B lymphocytes, which is at odds with the typical increased responsiveness of cells derived from WHIM patients (5–7). Such uncoupling of the expression level of CXCR4 and its chemotactic response has already been described also for B lymphocytes (25, 26). In general, CXCR4 is expressed throughout the whole B lymphocyte ontogeny where it fulfills different functions depending on the developmental stage (6, 27, 28). Also, mouse models underlined the key role of proper CXCR4 functioning in B-cell lymphopoiesis (29) and in the maintenance of the humoral response (30). We thus hypothesize that patient’ B cell dysfunctions could derive from an altered fine-tuning of the CXCR4/CXCL12 axis responsiveness as a consequence of anomalies in signal transducer(s), which has yet to be identified. Overall, these results reveal additional differences between our patient’s clinical and biological manifestations and those of the WHIM syndrome and suggest a novel IEI awaiting for the identification of a genetic defect in the CXCR4 signaling pathway that would account for the cutaneous and mucosal susceptibility to HPV-induced disease.

It is of interest that while our patient had normal frequency of circulating NK cells, an abnormal NK-cell subset distribution with a dramatic expansion of CD56^bright^ cells was observed. Patient’s CD56^bright^ cells, aside increased CD38 and NKp46 expression that is indicative of an enhanced activation status, presented an immature phenotype and efficient IFN-γ producing capability that normally distinguishes this NK-cell subset. In the peripheral blood of healthy subjects, the vast majority of circulating NK cells is CD56^dim^, while only a small percentage consists of CD56^bright^ cells that mainly reside in secondary lymphoid organs and are considered to be CD56^dim^ cell precursors (31). Of note, expansion of the CD56^bright^ subset has been observed in other disease settings including chronic infection and IEI (32, 33). As described in IEI cases, our patient showed a high frequency of immature CD16^-^NKG2A^+^NKG2C^-^CD57^-^ cells within the CD56 ^bright^ subset (33, 34). Moreover, CD56^bright^ cell expansion was paralleled by a decrease in both the frequency and the cytotoxic potential of CD56^dim^ cells. In keeping with more arguments in favor of CD56^bright^ cells being the precursors of CD56^dim^ cells (35), it can be postulated the presence in our patient of a partial maturation block at some step during differentiation from CD56^bright^ to CD56^dim^ cells. Intriguingly, our patient did not present NKG2C^+^ memory-like CD56^dim^ NK cells that are normally expanded in CMV-seropositive individuals as she was (22, 23). At present, however, we cannot discern whether our patient has a *bona fide* NK cell memory defect or belongs to the 4% of individuals with an *NKG2C^-^*/*NKG2C^-^* phenotype where memory-like NK cells should be identified by specific transcriptional reprogramming analysis (36). At any rate, the observed skewing of CD56^bright^ and CD56^dim^ subsets and the impaired cytotoxic potential of CD56^dim^ NK cells might have detrimental consequences in the responses towards viral infections as well as tumors.

Studies *in vitro* and in mouse models have shown that CXCR4/CXCL12 signaling is important for the development of NK cells, likely instructing NK-cell progenitors towards IL-15 niches formed by mesenchymal stem/progenitor cells (37–39). In particular, CXCR4 desensitization is required for the exit of most immature NK cells from the bone marrow, whereas CXCR4 appears to be dispensable for NK cell trafficking in lymph nodes (14). It is tempting to speculate that a shared molecular mechanism involving an abnormal CXCR4 signaling might have driven the altered differentiation pattern of B and NK cells in our patient, a hypothesis that deserves further investigation. In line with the important role of NK cells in the immune defense against viruses, a clinical hallmark shared by primary NK cell deficiencies is an unusual susceptibility to severe and/or recurrent viral infections, such as herpes virus and HPV infections (40). Therefore, a link may exist between the alteration of the NK-cell compartment and severe and persistent HPV pathogenesis in our patient. Consistent with this hypothesis, it has been reported the case of a woman affected by HPV-associated recalcitrant warts who had a dramatic CD56^bright^ cell expansion associated with low frequency of CD56^dim^ cells and impaired NK-cell cytotoxicity. Treatment with IFN-α resulted in warts disappearance and restoration of the NK cell function and subset distribution (41). More recently, it was also reported the case of a man with somatic reversion of an *IL2RG* germline mutation in T lymphocytes but not in NK cells, presenting recurrent warts and an intranasal HPV-related squamous-cell carcinoma and for whom NK cell included 31% of CD56^bright^ cells and displayed low cytotoxic function. Allogeneic hematopoietic-cell transplantation allowed normalization of the distribution on NK cell subpopulations and cytotoxic function and also the regression of HPV-related lesions (42). Moreover, killer immunoglobulin-like receptor (KIR) and HLA polymorphisms have been demonstrated to affect the individual susceptibility to various diseases including infections (43). In particular, different studies have shown the association of a specific KIR and/or HLA genotype with either protection against or increased risk for HPV-associated cervical neoplasia (44, 45). The occurrence that our patient might present KIR and/or HLA genotype that confers increased resistance of HPV-infected cells to cytotoxic responses needs to be investigated. The key role of CXCR4 in HPV16-induced oncogenesis in an experimental mouse model (46) and in the abnormal NK-cell distribution in a WHIM mouse model (14) support the idea that CXCR4/CXCL12 signaling could play a critical function in the control of HPV infection (13, 46, 47), with the important assistance of a balanced NK cell distribution and function (40). Turning to a common link governing B and NK cell abnormalities seen in our patient holds promise for the dissection of critical regulators involved in CXCR4/CXCL12 axis.

## Conclusion and Transparency Statement

Since the original description of our patient (17, 19), extended clinical monitoring as well as immunological and genetic investigations here presented have allowed further understanding of this novel IEI, underscoring the contribution of CXCR4/CXCL12 signaling pathway and of NK cells in host defense against HPV and providing new clues for targeted therapies.

## Data Availability Statement

The raw data supporting the conclusions of this article will be made available by the authors, without undue reservation.

## Ethics Statement

The studies involving human participants were reviewed and approved by the ethics committee of the Policlinico Tor Vergata, Rome Italy. The patients/participants provided their written informed consent to participate in this study. Written informed consent was obtained from the individual(s) for the publication of any potentially identifiable images or data included in this article.

## Author Contributions

GMFM and VM conceived the study. GMFM and MS provided clinical samples and patients’ clinical data. MD, SC, GDM, and VM-E performed experiments and analyzed the data. MD, GMFM, VM-E, and FB contributed to the study design and data interpretation. MD, GMFM, VM-E, and VM contributed to writing the manuscript. All authors have revised the manuscript and agreed to publish the final version.

## Funding

This work was supported by grants of the Association Laurette Fugain to FB and VM-E, and of the Italian Ministry of Health (Ricerca Corrente co-founded by Italian 5 x 1000 from Bambino Gesù Children’s Hospital) to MD.

## Conflict of Interest

The authors declare that the research was conducted in the absence of any commercial or financial relationships that could be construed as a potential conflict of interest.

## Publisher’s Note

All claims expressed in this article are solely those of the authors and do not necessarily represent those of their affiliated organizations, or those of the publisher, the editors and the reviewers. Any product that may be evaluated in this article, or claim that may be made by its manufacturer, is not guaranteed or endorsed by the publisher.
