# Supplementary material for: Case Report: Altered NK Cell Compartment and Reduced CXCR4 Chemotactic Response of B Lymphocytes in an Immunodeficient Patient With HPV-Related Disease
Source: Front Immunol. 2022 Jan 26;13:799564. doi: 10.3389/fimmu.2022.799564 (PMC8825485; doi:10.3389/fimmu.2022.799564)
Supplement: Supplementary file 1 [file DataSheet_1.docx]

# Materials and Methods

# Antibodies

# The following fluorochrome-labeled monoclonal antibodies (mAbs) were used in Figure 1: anti-CD3/FITC (clone HIT3a), CD4/PE-Cy5 (RPA-T4), CD8/Pacific Blue (RPA-T8), CD16/BV605 (3G8), CD19/AlexaFluor700 (HIB19), and CXCR4/APC (12G5) from BD Biosciences (Franklin Lakes, NJ, US); CD4/APC-Vio770 (REA623), CD56/APC-Vio700 (REA196), and CD57/PE-Vio700 (TB03) from Miltenyi Biotec (Bergisch Gladbach, Germany); CXCR5/PE (51505) from R&D Systems (Minneapolis, MN, US). In Figure 2 we used: CD3/AlexaFluor700 (UCHT1), CD56/PerCpCy5.5 (B159), CD16/BV510 (3G8), Perforin/BV421 (delta g9), IFN-γ/FITC (4S.B3), CD56/PerCp (MEM-188) from Thermo Fisher Scientific (Waltham, MA, USA); NKG2D(CD314)/PE (1D11), CD3/APC (UCHT1), CD16/APC-eFluor780 (CB16) from eBioscience (San Diego, CA, USA); CD107a/FITC (H4A3), DNAM-1(CD226)/FITC (11A8), NKp46/PE-Cy7 (9E2), KIR2DL1/S1/S3/S5/APC (HP-MA4), APC, KIR2DL2/L3/S2/APC (DX27), NKp46/PE-Cy7 (9E2) from BioLegend (San Diego, CA, USA); NKG2A(CD159a)/FITC (REA110), NKG2C (CD159c)/PE (REA205) from Miltenyi Biotech. In all experiments we used conjugated mouse IgG for isotype control staining from BD Biosciences.

# Cell surface expression of chemokine receptors

# Blood samples from the patient and from healthy donor(s) (HD) were collected in tubes with heparin, at the same time, maintained under same conditions and treated next day. Staining of different lymphocyte populations was performed with 100 μL of whole blood adding different mAbs combinations. After 30 min staining at 4°C, red blood cells were eliminated with OptiLyse C (Beckman Coulter, Brea, CA, US) following manufacturer’s instructions. Leukocytes were washed and analyzed with LSR-Fortessa cytometer (BD Bioscience). The relative expression level of chemokine receptors in patient cells was calculated as the ratio of receptor mean fluorescence intensity (MFI) in patient’s cells / MFI in HD’s cells x 100.

# Lymphocyte chemotaxis assay

# Whole blood samples were tested on a chemotactic migration assay using transwell inserts with 5 µm pores polycarbonate membrane (Corning Life Sciences, New York, NY, USA). In the lower chamber were placed 600 μL of CXCL12 (0, 10, 50 or 100 nM), CXCL13 (300 nM) or CCL19 (100 nM) diluted in RPMI 1640 medium with 0.1% human serum (chemokines were from from R&D Systems). Blood diluted in the same medium at a 1:4 ratio (100 μL) was placed in the upper chamber. When used, the CXCR4 specific inhibitor AMD3100 (Sigma-Aldrich, St. Louis, MO, US) was added in upper and lower chambers at 10 μM final concentration. After 4 hours of incubation, cells having migrated to the lower chamber were collected and stained with anti-CD3, anti-CD4, anti-CD8, anti-CD19, and anti-CD56 mAbs. Red blood cells were lysed and the entire sample was acquired by flow cytometry. Assay was performed with experimental duplicates. The percentage of cells of every lymphocyte population that responded to a chemokine was calculated as follows: {[(Number of cells recovered in the well with the chemokine) − (Number of cells recovered in a well with no chemokine)]/(Number of cells seeded in the upper chamber)} × 100.

# Analysis of NK cell subsets by flow cytometry

# PBMCs and serum were obtained by Ficoll separation of intravenous blood samples of the patient or HDs and cryopreserved until used. To assess viability, cells were stained with the LIVE/DEAD fixable NEAR-IR dead cell stain kit according to manufacturer’s protocol (Invitrogen Life Technologies, Carlsbad, CA, US). PBMCs were reacted with mAbs in staining buffer (1% bovine serum albumin in PBS) for 20 min at 4°C. For intracellular perforin staining, cells were treated with FOXP3 Fix/Perm Buffer Set (Biolegend) then incubated with mAbs for 30 min at room temperature. All immunolabeled cells were finally washed, resuspended in 1% paraformaldehyde, and acquired on a FACSCanto II (BD Biosciences) or Cytoflex (Beckman Coulter). Positive cell gating was set using fluorescence minus one control. The MFI was subtracted of the value obtained with isotype control antibody. Data analyses were performed using FlowJo software (TreeStar, Ashland, OR, US) or Kaluza (Beckman Coulter).

# NK-cell functional assays

# Flow cytometry-based cytotoxicity assays were performed using PBMCs as effectors (E) and the K562 erythroleukemia cell line or 721.221 lymphoblastoid EBV^+^ B cell line as targets (T). Cells were seeded at an E:T ratio of 10:1 in complete medium (RPMI 1640 medium with 10% fetal bovine serum, 0.2 mM L-glutamine, and 100 units/ml penicillin-streptomycin from Euroclone S.p.A, Pero MI, Italy, supplemented with anti-CD107a/FITC mAb or IgG_1_/FITC and incubated at 37°C for 6 h. After the first hour, monensin (Golgi stop, BD Biosciences, diluted 1:1500) and 10 µg/ml Brefeldin A (Sigma-Aldrich) were added to cultures. Finally, cells were immunolabeled and analyzed by flow cytometry to measure the frequency of CD107a^+^ cells within gated CD56^bright^ and CD56^dim^ NK cell populations. To measure IFN-γ production, PBMCs were plated at a concentration of 6x10^6^ PBMCs/mL in a 96-well U bottom plate in complete medium supplemented or not with cytokines (5 ng/mL IL-12, 100 ng/mL IL-15, 50 ng/mL IL-18; Peprotech, Rocky Hill, NJ, US) and cultivated for 20 hours. After the first hour, monensin and Brefeldin A were added to cultures. Finally, cells were harvested and stained in order to measure intracellular IFN-γ accumulation in gated NK cells by flow cytometry.
